# Typhoid Epidemic in Rockeville, Md.

**Published:** 1914-08

**Authors:** 


**Typhoid Epidemic in Rockeville, Md.,** L. L. Lumsden, Surgeon, U. S. P. H. S., Public Health Bulletin, No. 44. In a population of 1,200 28 cases of typhoid, beside some doubtful ones, occurred in January and February, 1914, following an imported case. The town is partly provided with water closets with septic tanks but mainly with unsanitary privies. Its water is derived from two 250-foot wells, beside private wells. All cases occurred in those using the town water. Water from one of the wells was shown to contain colon bacilli on various occasions and uranine deposited near the privy of the house in which the first case occurred, colored its water. The abatement of the epidemic seemed to be due to precautions in regard to excreta.

